# Though Lost to Sight, to Memory Dear

**Published:** 1882-11

**Authors:** Ruthven Jenkyns


### "THOUGH LOST TO SIGHT, TO MEMORY DEAR."

BY RUTHVEN JENKYNS.

And first published in the *Greenwich Magazine* one hundred and eighty-one years ago.

I.

Sweetheart, good-bye! the flutt'ring sail
Is spread to waft me far from thee,
And soon, before the fav'ring gale,
My ship shall bound upon the sea.
Perchance, all desolate and forlorn,
These eyes shall miss thee many a year,
But unforgotten every charm,
Though lost to sight, to mem'ry dear.

II.

Sweetheart, good-bye! one last embrace!
O, cruel fate! true souls to sever!
Yet, in this heart's most sacred place,
Thou, thou alone shalt dwell forever!
And still shall recollections trace
In Fancy's mirror, ever near,
Each smile, each tear, that form, that face
Though lost to sight, to mem'ry dear.

III.

Sweetheart, good-bye! though never more
The wave may bear me back to thee.
Though thrown upon some distant shore
By angry wind and surging sea,
My constant heart would still recall
Thy soft brown eyes, and browner hair,
And know thy touch, thy touch, thy all,
Though lost to sight, to mem'ry dear.

### THE CATS AND THE MONKEY.

A monkey, once weighing a nice piece of cheese,
(Which two cats had stolen to eat at their ease,
And wished it divided into parts quite fair,
So neither would have more nor less than his share),
Kept biting off pieces the right weight to find,
Till when it weighed even nothing was left but rind.

So often in lawsuits the clients discover
When the lawyers are paid there is nothing left over.

THERE was an increase in this country last year of 1,500 post-offices.

